# Effect of sampling face velocity on the ultrafine particle surface collection efficiency of a cellulose membrane filter and a cellulose-glass fiber filter for environmental airborne radioactivity monitoring

**DOI:** 10.1093/rpd/ncae191

**Published:** 2024-11-14

**Authors:** Mizuki Kiso, Manaya Taoka, Aoi Sampei, Hiroki Hashimoto, Yuki Abe, Yuki Oda, Yasutaka Omori, Ryohei Yamada, Masahiro Hosoda, Chutima Kranrod, Tetsuo Ishikawa, Shinji Tokonami

**Affiliations:** Hirosaki University Graduate School of Health Sciences, 66-1 Honcho, Hirosaki, Aomori 036-8564, Japan; Hirosaki University Graduate School of Health Sciences, 66-1 Honcho, Hirosaki, Aomori 036-8564, Japan; Hirosaki University Graduate School of Health Sciences, 66-1 Honcho, Hirosaki, Aomori 036-8564, Japan; Hirosaki University Graduate School of Health Sciences, 66-1 Honcho, Hirosaki, Aomori 036-8564, Japan; Hirosaki University Graduate School of Health Sciences, 66-1 Honcho, Hirosaki, Aomori 036-8564, Japan; Hirosaki University Graduate School of Health Sciences, 66-1 Honcho, Hirosaki, Aomori 036-8564, Japan; Institute of Radiation Emergency Medicine, Hirosaki University, 66-1 Hocho, Hirosaki, Aomori 036-8564, Japan; Institute of Radiation Emergency Medicine, Hirosaki University, 66-1 Hocho, Hirosaki, Aomori 036-8564, Japan; Hirosaki University Graduate School of Health Sciences, 66-1 Honcho, Hirosaki, Aomori 036-8564, Japan; Institute of Radiation Emergency Medicine, Hirosaki University, 66-1 Hocho, Hirosaki, Aomori 036-8564, Japan; Institute of Radiation Emergency Medicine, Hirosaki University, 66-1 Hocho, Hirosaki, Aomori 036-8564, Japan; Department of Radiation Physics and Chemistry, Fukushima Medical University, 1 Hikarigaoka, Fukushima 960-1295, Japan; Institute of Radiation Emergency Medicine, Hirosaki University, 66-1 Hocho, Hirosaki, Aomori 036-8564, Japan

## Abstract

Surface collection efficiency (SCE) of a cellulose membrane filter (CMF) and a cellulose-glass fiber filter used in environmental monitoring for alpha-emitting radionuclides from nuclear facilities and natural radioactivity sources was evaluated for particles in the size range of 0.03–0.1 μm at different levels of face velocity. The SCE of the CMF was higher than that of the cellulose-glass fiber filter, and only the membrane filter showed the dependence of SCE on the particle size at higher face velocity. The use of the CMF at higher face velocity in environmental radioactivity monitoring leads to measurements of the background alpha spectrum with more degradation under the changing particle size condition in the atmosphere. Consequently, that fact needs to be taken into account, along with the expected particle size distribution and concentration of the airborne radioactivity being sampled, when selecting a face velocity to achieve the best possible detection limit.

## Introduction

Aerosol monitors with continuous or intermittent air sampling are installed around nuclear facilities to detect accidental release of artificial radionuclides from the facilities. The airborne radionuclides are typically collected on filters, and subsequently the radioactivity of the collected radionuclides is measured by alpha or gamma spectroscopy [1]. The sampling is performed for aerosols with diameters of a few micrometers for face velocities of approximately 30–110 cm s^−1^ to detect trace amounts of the artificial radionuclides [2–4]. Various considerations are made for selecting filters for the aerosol monitors such as collection efficiency, pressure drop during sampling, and ease of handling in the chemical analysis. Among these points, surface collection efficiency (SCE) is particularly important in detecting alpha-emitting radionuclides. Energy loss of alpha particles passing through filters with low SCE causes poor identification of radionuclides and underestimation of their activity concentrations. In addition, the presence of alpha-emitting natural radionuclides such as radon progeny affect the detection of the artificial radionuclides. Low SCE causes spectrum tailing of alpha particles emitted from the natural radionuclides (e.g. ^218^Po, 6.003 MeV; ^214^Po, 7.687 MeV) toward the lower energy side, which causes an increase in detection limits of artificial radionuclides such as ^239^Pu (5.157 MeV) or ^241^Am (5.486 MeV).

The SCE depends on filter types, and face velocity and particle size of sampled aerosols. The SCE of cellulose-glass fiber filters is generally lower than that of membrane filters [5]. For glass fiber filters, the SCE was reported to increase with aerosol size and face velocity of sampling in the ranges of 0.55–3.07 μm and 50–200 cm s^−1^, respectively [6]. A similar relationship was found for membrane filters in the aerosol size and face velocity ranges of 0.03–0.8 μm and 1.5–18.4 cm s^−1^, respectively [7–9]. However, these relationships are not applicable to quantification of the SCEs of filters for smaller particles (≤ ~0.1 μm in diameter) at a higher face velocity (> tens of centimeters per second). The SCEs in these ranges affect detection ability of the artificial radionuclides because radon progeny of less than 0.1 μm in diameter dominate the number size distribution, but not the mass distribution, in the atmosphere [10].

The present study evaluated the dependence of the SCE of a cellulose membrane filter (CMF) and a cellulose-glass fiber filter for particle sizes less than 0.1 μm at different face velocities up to about 70 cm s^−1^.

## Materials and methods

### Experimental setup

**Figure 1:**
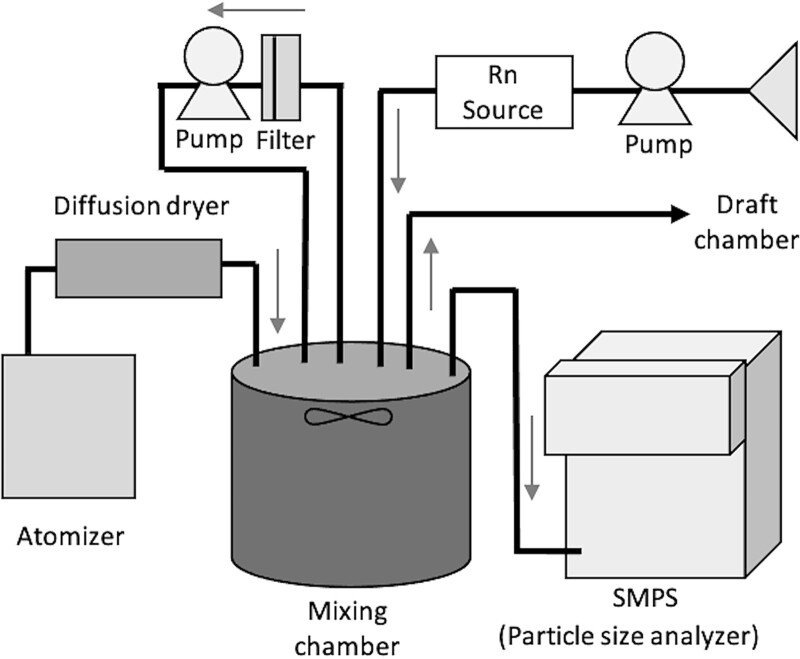
Experimental setup using a radon exposure chamber to determine the SCE of filters.

The SCEs of the filters was examined using a radon exposure chamber at Hirosaki University (Fig. 1) [11]. Radon progeny was mixed with NaCl particles sprayed using an atomizer (Model 3079; TSI Incorporated, USA) to produce radioactive aerosols. Their size distributions were measured every 3 min continuously throughout the collection time using a scanning mobility particle sizer (Model 3034; TSI Incorporated). Then, the count median diameter (CMD) and the geometric standard deviation (GSD) were calculated from the size distribution summed over the measuring period. Since the aerosol particle size distribution can be approximated by a log-normal distribution, the CMD is equivalent to the geometric mean diameter. The generated aerosols had poly-dispersed size distributions with CMD values of 0.03–0.1 μm (GSD 1.5–2.0) at 10–10 000 ppm of an NaCl solution in the atomizer. Activity median diameter (AMD) was converted using the following formula [12]:

*AMD* = *CMD* exp (2 ln^2^*GSD*).

The filters examined in the present study were a coated CMF (pore size, 2.0 μm; diameter, 25 mm; Y020A025A; Advantec Toyo Kaisha, Ltd., Japan) and a cellulose-glass fiber filter (diameter, 25 mm; HE-40T; Advantec Toyo Kaisha, Ltd., Japan). The CMF was selected because it has high stability of flow rate and it is applicable to chemical analysis to confirm activity of detected artificial radionuclides [5]. The HE-40T has been widely used for environmental radioactivity monitoring in Japan [13]. Each filter type was fixed into an in-line filter holder (effective area, 3.5 cm^2^) to collect the radioactive aerosols. The sampling was performed at four face velocity levels of 4.8, 23.8, 47.6, and 71.4 cm s^−1^. After the sampling was completed and the collected ^218^Po (half-life 3.1 min) was allowed to decay for a minimum of 30 min so that less than 0.1% of the initial ^218^Po activity remained, the filter sample radionuclides were analyzed for 60 min using a silicon semiconductor detector PIPS (CAM 490 AM; CANBERRA Industries Inc., USA) to obtain the energy spectra of ^214^Po. The spectrum analysis was made under a vacuum condition, to avoid self-absorption by the air. In addition, the distance between the detector and filter was set to 25 mm to reduce occurrence of oblique incidence of emitted alpha particles to the detector surface. During this measurement, the total accumulated counts of ^218^Po were approximately 10^3^–10^4^ counts, and the average relative error for SCE determined from the counts was 8% for CMF and 25% for HE-40T. The higher face velocity and larger particle size led to the higher accumulated counts.

### Calculation of SCE

**Figure 2:**
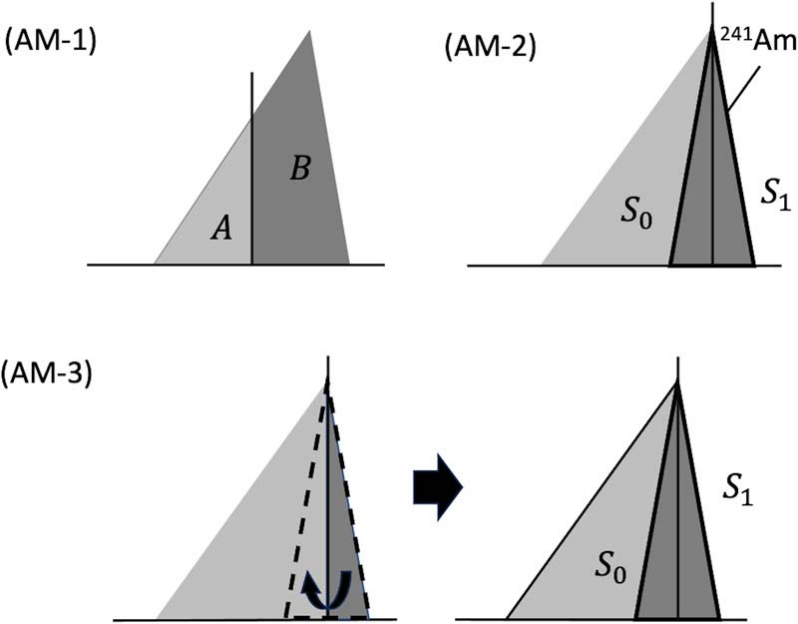
Conceptual diagrams for calculating the SCE of filters. Vertical and lateral axes represent counts and channel numbers of the alpha particle spectrum, respectively. The figure was modified from Tamakuma et al. [5].

The analytical method for SCE has not been established yet and, therefore, three methods were used to observe its relative changes with respect to aerosol size and face velocity. The methods calculated the SCE of the filters using the energy spectrum of ^214^Po (Fig. 2) [5]:

(AM-1) The alpha spectrum was divided into two regions with respective counts *A* and *B* of 2.00–6.68 and 6.68–8.50 MeV including the alpha energy in ^214^Po. The SCE was calculated by the counts *B* to total counts *A* + *B*.

(AM-2) The SCE was determined by using the alpha energy spectrum of an ^241^Am planar source. Its spectrum shape was approximated as being the same shape as that formed by alpha emitters deposited on the filter surface. The ^214^Po spectrum was imposed on the ^241^Am spectrum to obtain the counts (*S*_1_) caused by the surface deposition. The SCE was calculated as the ratio of *S*_1_ to all counts (*S*_0_ + *S*_1_) of the ^214^Po spectrum.

(AM-3) The energy spectrum was approximated as a normal distribution depending on the energy resolution of the detector. The spectrum tail was folded from the peak energy to the lower energy side to approximate the normal distribution. The SCE was determined in the same way as for method AM-2.

## Results

**Figure 3:**
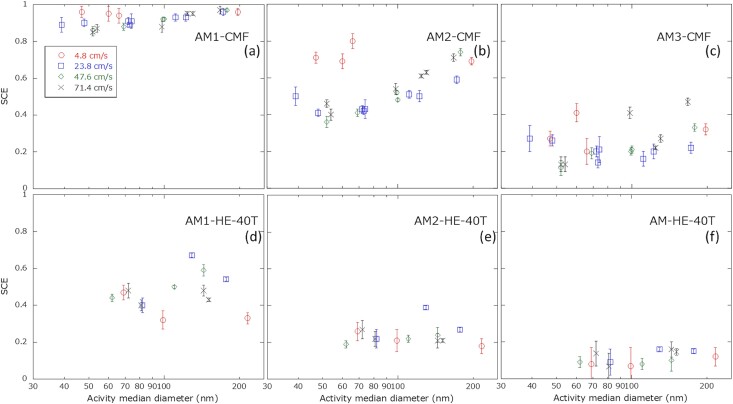
Scatter plots of SCE against activity median diameter of radon progeny. Panels (a)–(c) present the SCEs for CMF determined by the analysis methods AM-1 to AM-3, respectively. Panels (d)–(f) present the SCEs for HE-40T determined by AM-1 to AM-3, respectively.

Figure 3 shows scatter plots of the SCEs against activity median diameters of radon progeny for the CMF and HE-40T. The SCEs of CMF were twice as high as those of HE-40T. In the CMF results (Figs. 3a–c), no clear dependence of the SCE on activity median diameter was seen at face velocities of 4.8 and 23.8 cm s^−1^, whereas the SCE increased with activity median diameter at face velocities of 47.6 and 71.4 cm s^−1^. In contrast, in the HE-40T results (Figs. 3d–f), no clear dependence of the SCE on the activity median diameter in the range of 0.03–0.1 μm was seen for any of the examined face velocity levels.

## Discussion

According to infiltration theory, particle collection onto filters follows Brownian diffusion behavior for the filter surface, interception for pore openings, and inertial impaction for the filter surface [7–9]. The Brownian diffusion is dominant among smaller particles (up to a few tens of nanometers) and its effect decreases with increasing particle size. On the other hand, the effects of interception and inertial impaction increase with increasing particle size and they are dominant among larger-size particles (several tens of nanometers to 100 nm). In the range of a few to several tens of nanometers in particle size, the transition region of these three collection mechanisms [7], the particle collection efficiency reaches a minimum. The minimum values vary depending on the face velocity, and a higher face velocity contributes to penetration of particles through filters in this particle size range.

The SCEs of CMF increased with increasing activity median diameter at the higher face velocities of 47.6 and 71.4 cm s^−1^ (Figs. 3b and c). This result indicated interception and inertial impaction rather than Brownian diffusion were dominant in particle collection in this higher face velocity range. Occurrences of Brownian diffusion was fewer due to the shorter residence time near the filter surface [9]. On the other hand, at the face velocities of 4.8 and 24 cm s^−1^, the SCEs were constant throughout the examined activity median diameter range (Fig. 3). According to infiltration theory, minimum SCE values are seen when the collection mechanism transits from Brownian diffusion to interception and inertial impaction. In addition, a lower face velocity leads to less penetration of particles through filters in the transition region [7,9]. Thus, the experimental results in these two cases implied that the SCEs did not change largely throughout the particle size range even including the transition region due to the lower face velocity.

The SCE of the HE-40T fiber filter did not clearly depend on particle size and face velocity. To the best of the authors’ knowledge, such a relationship has not been reported in this particle size range except for the present case. Broadening the particle size range may help to determine the dependence and clarify the surface collection mechanisms.

The present study focused on collection of radon progeny with the activity median diameter of < 0.1 μm typically seen in atmospheric aerosols [10], unlike artificial radionuclides with a 1-μm activity median diameter accidentally released from nuclear facilities into the environment. This is because detection of accidental release of the artificial radionuclides is affected by the presence of radon progeny. Use of low-SCE filters causes the lower energy tail of ^218^Po (6.0 MeV) to extend into the range of the artificial radionuclides (3–5.7 MeV, such as ^239^Pu, ^241^Am). No dependence of SCEs on the particle size of the aerosols in the atmosphere is desirable to reduce false detection of the artificial radionuclides caused by fluctuations in the background spectrum of ^218^Po.

The SCE of the CMF depended on the activity median diameter at the higher face velocities. This means that the background spectrum will fluctuate following changes in the particle size of ambient aerosols. Therefore, when using the CMF, a low sampling face velocity can suppress the spectral variation due to particle size and, consequently, prevent the false detection.

On the other hand, the SCE of the HE-40T did not clearly depend on activity median diameter in the examined range. However, it was one half the SCE for the CMF. In order to distinguish signals of artificial radionuclides from background of natural radionuclides in the energy spectrum, a high SCE that provides a sharp energy spectrum is required. Therefore, it is important to have a high SCE as well as no dependence of SCE on particle sizes less than 0.1 μm.

The face velocity for the CMF in environmental radioactivity monitoring should be optimized to ensure the best possible detection limit. From the viewpoint of reducing background spectrum fluctuations, this study suggests that a relatively low face velocity is desirable to lower the dependence of SCE on particle size. The increase in the filter diameter can realize the surface velocity reduction, whereas it can increase oblique incidence and cause spectral degradation. From the viewpoint of collecting bigger particles (~1 μm in diameter) possibly released from nuclear facilities, the collection efficiency increases as the face velocity increases [14]. The face velocity is optimized in reducing the background spectrum fluctuation as well as in increasing bigger particle collection efficiency. It is noted that the aforementioned discussion is applicable only to new filters, since dust loads can cause energy absorption that changes both the energy spectrum and collection efficiency.

The CMF is a membrane filter with a fibrous structure. However, the SCE-particle size relationship for the CMF was similar to that for nuclepore filters reported in previous studies [7–9] rather than for the HE-40T. This finding should be examined further in future work to clarify the infiltration mechanism of ultrafine particles. The present study was carried out for radioactive aerosols with NaCl particles in the restricted range of around 0.03–0.1 μm. This particle size range covers atmospheric aerosols from natural sources [10] but not the full range of environmental aerosols from sources such as nuclear facilities and is therefore only partially useful for setting the face velocity. In addition, this study may contain uncertainties in the results of conversion from CMD to AMD.

## Conclusion

This study evaluated the dependence of the SCE of a coated cellulose membrane filter (CMF) and a cellulose-glass fiber filter (HE-40T) on particle size (activity median diameter) and face velocity of sampling. The SCE of the HE-40T had no dependence on the activity median diameter of less than 0.1 μm. On the other hand, the SCE of the CMF at high face velocities (48 and 71 cm s^−1^) depended on the activity median diameter. The CMF showed higher SCEs and the spectrum was not degraded much compared to the HE-40T, making it easy to discriminate between nuclides. Consequently, face-velocity-dependent SCE factors must be taken into account along with the expected particle size distribution and concentration of the airborne radioactivity being sampled when selecting a face velocity to achieve the best possible detection limit.
